# Nutrition Society of New Zealand Annual Conference Held in Queenstown, New Zealand, 28–29th August 2014

**DOI:** 10.3390/nu6114731

**Published:** 2014-10-29

**Authors:** Sheila Skeaff

**Affiliations:** Department of Human Nutrition, University of Otago, PO Box 56, Dunedin 9054, New Zealand; E-Mail: sheila.skeaff@otago.ac.nz; Tel.: +64-3-479-7944; Fax: +64-3-479-7958

## 1. Preface

The annual conference and scientific meeting of the Nutrition Society of New Zealand took place in Queenstown, New Zealand from 28th–29th August, 2014. The meeting was part of Queenstown Research Week, established in 1991, which includes the Queenstown Molecular Biology Meeting, the New Zealand Medical Sciences Congress, and the Australasian Society of Clinical and Experimental Pharmacologists and Toxicologists New Zealand Scientific Meeting. Various societies take part in the different meetings; this was the first year that the Nutrition Society of New Zealand was included. The theme of the Nutrition Society of New Zealand in 2014 was “Balancing Views”. The plenary session “Weighing up the Evidence: Nutrition Controversies” was provided to address ongoing debate in New Zealand about the role of saturated fat in chronic disease, particularly cardiovascular disease. 

## 2. Summary of Scientific Presentations

### 2.1. Changes in Portion Sizes of Snack Foods Consumed by New Zealand Adults August, C.R.; Smith, C.; Fleming, E.A.; Parnell, W.R.

Background: Increases in food portion sizes have been suggested as one factor contributing to rising obesity rates. To date no studies have been published around changes in portion sizes consumed by NZ adults. Therefore, the primary objective of this study was to compare portion sizes per eating occasion of selected snack foods between the 1997 National Nutrition Survey (NNS97) and the 2008/2009 Adult Nutrition Survey (ANS08/09). 

Methods: Both the NNS97 (*n* = 4636) and ANS08/09 (*n* = 4721) collected dietary information using computer based multiple pass 24-h diet recall. Commonly reported snack foods were selected for comparison including soft drinks, biscuits, cakes, crackers, muffins, scones, slices, puddings, chocolate, sweets and confectionary, ice-cream, muesli bars and potato crisps. 

Results: Linear regression models were used to compare the mean portion size per eating occasion in grams between the surveys. Significant increases were found in the portion size of biscuits reported by males (5.3 g, *p* = 0.048) and females (4.2 g; *p* = 0.008), ice-cream for males (24.2 g, *p* = 0.005) and sweets in females (10.3 g; *p* = 0.044). The portion sizes of slices reported by males decreased between the surveys for males (20.3 g; *p* = 0.033), as did the portion size of muffins reported by females (21.0 g; *p* = 0.014). Among this group of foods there were some increases in the portion sizes reported, balanced by decreases for other food groups. 

Conclusions: This research provides valuable information on both changes in portion sizes over time in addition to documenting typical portion sizes consumed. 

The NZ Ministry of Health funded the surveys, which were undertaken collaboratively with the University of Otago. The NZ Crown is the owner of the copyright for the survey data. The results presented are the work of the authors. 

### 2.2. Vitamin D Status of Māori and non-Māori New Zealanders of Advanced Age from LiLACS NZ Cohort Study Bacon, C.J.; Kerse N.; Moyes, S.A.; Teh, R.; Hayman, K.J.; Dyall, L.; Kepa, M.; Bolland, M.J.

Background: Low vitamin D status is linked to falls and fractures in advanced age, though less data exist from cohorts in their 80s compared to younger groups. 

Methods: Levels of 25-hydroxyvitamin D [25(OH)D] and its cross-sectional determinants were assessed in 209 Māori (aged 80–90 years) and 357 non-Māori (aged 85 years) living within defined regional boundaries in the Bay of Plenty/Lakes District. 

Results: Levels in Māori (59 ± 4 nmol/L; mean ± 95% CI) were lower than those in non-Māori (75 ± 3 nmol/L; *p* < 0.001), a difference maintained when 25(OH)D was adjusted for the day of the year collected. High levels (>100 nmol/L) were present in 15% of individuals, with six individuals >150 nmol/L. Vitamin D supplementation was reported by 98 (18%) of participants, including a greater proportion of women (24%) than men (11%; *p* < 0.001) and of non-Māori (24%) than Māori (7%; *p* < 0.001), and half (49%) were taking high oral doses ≥25 µg/day (1000 IU/day). Only two individuals who took vitamin D were insufficient (<50 nmol/L). Seasonally-adjusted 25(OH)D was positively correlated with physical activity (rho = 0.09; *p* = 0.03), and inversely correlated with body mass index (rho = −0.16) and percent fat (rho = −0.14; *p* < 0.001 for both). For those not taking vitamin D, these variables did not independently predict seasonally-adjusted 25(OH)D. 

Conclusions: Vitamin D status in this group of New Zealanders of advanced age is high compared to previous studies of older adults, and is associated with use of high-dose oral vitamin D. Levels are substantially higher in non-Māori, compared to Māori, and may in part reflect inequitable supply of oral vitamin D.

### 2.3. The Effects of Regular Nut Consumption on Nutrient Profiles and Food Group Patterns in Comparison to Other Energy Dense Snack Foods Brown, R.; Pearson, K.; Chisholm, A.; Tey, S.L.

Background: Regular nut consumption is associated with a reduction in cardiovascular disease risk, partly due to improvements in diet quality. How individuals modify their intakes when consuming nuts compared to other snacks is largely unknown. We therefore examined the effects of nut consumption in comparison to other energy-dense foods on energy compensation, nutrient displacement and food group patterns.

Methods: This was a 12-week randomised, controlled, parallel study with four arms: 1100 kJ/day for each of hazelnuts (42 g), chocolate (50 g), potato crisps (50 g), or no added snack (control). Food records, body composition, and physical activity were measured at baseline and week 12 in 102 participants. 

Results: There were no significant changes in body composition and physical activity between groups. However, significant improvements in diet quality were observed in the hazelnut group. Intakes of monounsaturated fat, polyunsaturated fat, and vitamin E were significantly higher (all *p* < 0.05), whereas saturated fat and carbohydrate were significantly lower (both *p* < 0.05) in the hazelnut group compared to the other groups at week 12. When foods were categorised as snacks or meals, changes in nutrient intake were apparent only for snacks. There were no significant between group differences for energy compensation and displacement for nutrients (all *p* ≥ 0.132), with the exception of fibre. Changes in food group intake were largely limited to food groups to which the snacks were allocated. 

Conclusions: Results show that regular nut consumption significantly improves nutrient profiles, without adversely affecting body composition. Also, nutrient changes occur at the snack level and are not extended to meals.

### 2.4. A Pilot Study of the Nutritional Status of Patients Undergoing Haemopoietic Stem Cell Transplantation Burrows, L.; Price, M.; Butler, A.; Skeaff, S.

Background: The nutritional status of patients undergoing haemopoietic stem cell transplants (HSCT) is often compromised as patients often experience adverse side effects from treatment affecting dietary intake. The current research frequently reports compromised nutritional status in HSCT patients; however few of these studies have measured diet, an essential component of nutritional assessment. The primary aim of this study was to investigate the nutritional status of patients undergoing HSCT using dietary, anthropometric, biochemical and clinical measures. 

Methods: This pilot study was based in the bone marrow transplant unit (BMTU) at Christchurch Hospital. The study aimed to follow 10 participants from one month prior to HSCT until discharge from hospital. Participants completed weighed three-day diet records, patient guided-subjective global assessment (PG-SGA), anthropometric measures, twenty-four hour urine collections and blood tests on two occasions (pre-HSCT and post-HSCT). 

Results: Eight participants who underwent a planned autologous-HSCT completed the study. Post-HSCT, there was a decrease in energy (9051 ± 2662 kJ/day to 3330 ± 1337 kJ/day; *p* < 0.01) and protein intake (89 ± 26 g/day to 35 ± 20 g/day; *p* < 0.01). Macronutrient and micronutrient intakes no longer met EAR, RDI or BMT recommendations. Post-HSCT, percentage weight loss was >2%, indicating severe weight loss. The PG-SGA scores increased from 3 to 20, and the most common reported symptoms were nausea, vomiting and diarrhoea. 

Conclusions: This pilot study found the nutritional status of HSCT patients was compromised highlighting the importance of monitoring HSCT patients post-discharge. Further research is required on the nutritional status of HSCT patients. 

### 2.5. Improving Glycaemic Control with Manuka Honey-Based Products Chepulis, L.M.; Francis, E.

Background: The antimicrobial and anti-inflammatory properties of Manuka honey are well recognized; however, evidence suggests that it may also have an improved glycaemic response compared with other sugars. Complexing with α-cyclodextrin (a poorly digested five glucose ring unit) may improve glycemic control further. 

Methods: Two clinical studies were carried out to assess the glycaemic profile of raw and complexed Manuka honey. (1) Five high-methylglyoxal honey samples from different regions of New Zealand were tested for glycemic index (GI) in 10 healthy volunteers in a single-blinded, randomised study. Participants were fed honey or glucose containing 25 g of available carbohydrate in 200 mL water. The area under the blood glucose curve was measured over two hours. (2) The GI of Manuka honey was compared with α-cyclodextrin-complexed Manuka honey and glucose using 10 new subjects and matched methodology. 

Results: (1) All five honeys had moderate GI values (54–59) despite all samples containing >70% w/w monosaccharides. (2) The GI of Manuka honey was reduced substantially when complexed with α-cyclodextrin (18 *vs.* 55); however, 45 g (dose required to deliver 25 g available carbohydrate) was associated with gastrointestinal upset in several participants.

Conclusions: Both raw and cyclodextrin-complexed Manuka honey have potential as dietary sugar replacements. Further studies are needed to evaluate the levels of cyclodextrin-complexed Manuka honey that can be consumed to ensure good levels of gastrointestinal tolerance. 

### 2.6. The Scientific Substantiation of General Level Health Claims in New Zealand Crowe F.L.; Miller, J.; Reid, J.; Hathaway, S.

Background: In 2013, a new food standard (Standard 1.2.7—Nutrition, Health and Related Claims) was adopted in Australia and New Zealand and provides a scientifically robust regulatory pathway for food industry to make nutrition and health claims. Within Standard 1.2.7 the health claims are divided into general level and high level health claims; high level health claims refer to an effect on a serious disease or a biomarker for a serious disease, and general level health claims refer to a health claim that is not high level. The new standard lists a large number pre-approved health claims that can automatically be used by industry if they meet general nutrition criteria and conditions specific to the claim. The unique element of Standard 1.2.7 is the provision for industry to self-substantiate their own general level health claim if, on the basis of a systematic review, they can conclude that there is a convincing cause and effect relationship between the food and the effect on health. The Ministry for Primary Industries (MPI) has an essential role in reviewing the evidence base provided by industry to support new food health relationships. Given that foods contribute substantially to the growth of New Zealand exports, it is crucial that for both New Zealand industry and MPI’s global reputation and credibility, that this process of substantiation is scientifically robust. MPI is also committed to providing whatever technical assistance it can to help industry develop substantiated health claims.

### 2.7. Measuring Stakeholder Attitudes to Help Localise a University College’s Food Service Greer, J.; Mirosa, M.; Spence, H.

Background: Foodservices impact many consumers and businesses within their communities with their practices. This is most pertinent when bringing in new interventions. Successful change requires forethought and understanding stakeholder attitudes.

Methods: This study’s research question was “What are the dominant stakeholder discourses about local food and how might an understanding of these be used to help management localise the foodservice?” The study setting was in a traditional university college foodservice in Otago, New Zealand. The foodservice provided three meals a day for 187 first year students. Students, foodservice staff, management staff and food suppliers were identified as key stakeholders (*n* = 47).

Results: Q methodology, a type of inverted factor analysis, was used to measure dominant shared viewpoints (factors). The four factors that emerged from the data were named “The Leaders, “The Idealists”, “The Globalist” and “The Individualists”. “The Leaders” were informed and proactive but realistic about what they wanted to implement. They could be approached initially to do the groundwork to make local food a reality in their foodservice. While all these discourses were supportive of local food they differed in how they defined “local” and which initiatives and policy changes they found appealing. Results and recommendations will detail these to contribute to the foodservice literature. They will give an acceptable definition of ‘local’ for different food items, identify barriers to localisation and discuss how they could be resolved by working within and between stakeholder groups.

Conclusion: The results will be used to determine an acceptable definition of ‘local’ for different food items, identify barriers to localisation and discuss how they could be resolved by working within and between stakeholder groups.

### 2.8. Effect of Processing on the Oxalate Concentration of Com Hen and Canh Chua Bac Ha, Two Local Dishes Prepared from the Petioles of Taro Grown in Central Viet Nam Hang, D.T.; Loc, N.T.; Tra, T.T.T.; Tuan, L.M.; Savage, G.P.

Background: Petioles of Mon Ngot (Sweet Taro, *Colocasia esculentia*) grown in Thua Thien Hue Province in Viet Nam are an important ingredient in Com Hen, a popular local dish. During the preparation of the petioles the tough, fibrous outer skin, known to cause itchy throats, is removed. 

Methods: This experiment investigated the effect of removing the outer skin on the oxalate composition of the washed, cooked stems. The petioles were cooked for 10, 15 and 20 min to encourage increased leaching of soluble oxalates into the cooking water. Preparation of the related dish Canh Chua Bac Ha involves the natural fermentation of the petioles with lactobacillus to give a more even taste. Informal tastings after fermentation have reported a reduction in a sharp taste associated with high oxalate foods.

Results: Removing the outer skin of the petioles led to an 18% reduction in the soluble oxalate content of the raw stems. Boiling the whole peeled petioles resulted in a 42% reduction in soluble oxalate after 10 min when compared to the original raw petioles and a reduction of 60% after boiling for 15 and 20 min. Fermentation of the whole petioles for 48 h reduced soluble oxalate by 36% but there was only a 25% reduction after fermentation for 24 and 72 h. 

Conclusion: Taro petioles constitute between 30% and 40% of the final local dishes. These dishes do not include foods high in calcium that would bind soluble oxalate. This emphasises the importance of adequate processes for removing soluble oxalates prior to consumption.

### 2.9. The Prevalence of Low Vitamin B12 Status in People with Type 2 Diabetes Receiving Metformin Therapy in New Zealand—A Clinical Audit Haeusler S.; Parry-Strong A.; Krebs J.D.

Background: Metformin, the most common hypoglycaemic agent used in type 2 diabetes, is associated with reduced vitamin B12 status. This cross sectional observational study determines the prevalence of low Vitamin B12 status in people with Type 2 Diabetes on Metformin therapy in both primary and secondary care in New Zealand. 

Methods: All eligible patients seen in a secondary-care clinic over a 15-month time frame were screened for Vitamin B12 status (*n* = 347). Additionally, patients from four primary health care providers were identified using metformin prescription data and offered the chance to participate in the audit. 

Results: Prevalence of serum Vitamin B12 <200 pmol/L was 18.7%. Positive correlations were observed between B12 status, age and dosage and duration of Metformin treatment. Maori and Pacific Islanders had higher mean serum B12 values than European but no difference in prevalence of low B12 status. 

Conclusions: Low serum B12 level is a common occurrence in people with Type 2 diabetes treated with Metformin. Whether this is solely due to Metformin or influenced by age and dietary factors is unclear. Systematic screening in those receiving Metformin is advisable, particularly for patients older than 50 years. 

### 2.10. Bioactivity of Phytochemicals from Carrots Depends on the Form of Carrots Consumed: Effect of Juiced and Diced Carrots on Platelet Aggregation Herath, T.D.; Morgenstern, M.; Massarotto, C.; Ansell, J.

Background: Carrots (*Daucus carota* L), contain a variety of phytochemicals including polyacetylenes such as falcarinol, which is known to inhibit platelet aggregation, thus reducing blood-clotting and maintaining healthy blood flow. A preliminary human trial demonstrated the inhibitory effects of carrot on platelet aggregation in the presence of the agonist, arachidonic acid (AA). 

Methods: A non-randomised single group study was carried out, to investigate whether observed bioactivity was affected by the form in which it was consumed. Healthy volunteers (*n* = 10) had a standardised light breakfast, provided a baseline blood sample and then consumed 100 g of freshly diced or 100 mL of freshly juiced carrot (including pulp). Further blood samples (10 mL) were collected at 2, 5, 8 and 24 h after breakfast. 

Results: Some subjects showed a decrease in % platelet aggregation triggered by AA, while others had no reduction in platelet aggregation. This suggests differential responsiveness to this specific platelet aggregation pathway induced by AA. The responders showed significantly (*p* < 0.05) reduced platelet aggregation 2 h after consumption of juiced carrots (9.8%) compared with their response to diced carrots (70.5%). 

Conclusion: It is possible that there was more rapid digestion and absorption of nutrients from the carrot juice in the gastrointestinal tract, as a result of the plant cell structure being disrupted by the juicing process compared with the more intact plant cell structure of the diced carrots. Thus the possibility that release of bioactive compounds from carrots can be enhanced by juicing should be further explored.

### 2.11. Saturated Fat Still Causes Coronary Heart Disease: The Current ‘Controversy’ Has no Basis Jackson, R.T.

The epidemiological evidence linking diets high in saturated fats to increased risk of coronary heart disease has been scientifically convincing for many years and this evidence has continued to accumulate. The recent surge in local and international media attention given to the saturated fat sceptics is not based on any new evidence that conflicts with what is already known. Rather, it appears to be motivated by the hypothesis that international recommendations to reduce fat consumption have led to the substitution of fat in the diet with carbohydrates which in turn has led to the global obesity epidemic. The advocates of this hypothesis appear to be arguing that all fats are good for health and all carbohydrates are bad. This simplistic argument is wrong on almost every count. This keynote presentation will focus on the evidence demonstrating why the overwhelming majority of experts in this field remain convinced that diets high in saturated fat are causally linked to coronary disease and that substituting dietary saturated fats with poly- and monounsaturated fats will reduce the risk of coronary disease. 

### 2.12. The Effects of a Vitamin D, Omega 3, Co-enzyme Q10, Zeaxanthin, Lutein and Astaxanthin Supplement (Lester’s Oil) on Healthy People: Preliminary Results Laing, B.; Ellet, S.; Marlow, G.; Han, D.Y.; Jesuthasan, A.; Ferguson, L.R.

Background: Diet is a key component in the disease susceptibility of individuals. Reducing inflammation especially in people with inflammatory disorders is thought to decrease disease susceptibility. The aim of this trial was to investigate the effects of a dietary supplement which contains Vitamin D, Omega 3, Co-enzyme Q10, Zeaxanthin, Lutein and Astaxanthi on inflammatory markers in healthy people. 

Methods: The cross over trial was double blinded, randomised and placebo controlled. The study population (*n* = 30) was recruited from Auckland, New Zealand. The intervention or placebo was for 28 days, followed by a washout of 28 days followed by the placebo or intervention for 28 days. In this preliminary analysis blood samples were measured for C-Reactive protein (CRP), HDL, LDL, Triglycerides and cholesterol levels. A quality of life questionnaire, height and weight were also assessed. 

Results: Analysis of these measures found significant differences between the intervention and placebo groups for CRP (*p* < 0.0288) HDL (*p* < 0.0019) and triglycerides (*p* < 0.0091). 

Conclusion: In this preliminary analysis the supplement was shown to be effective in reducing key inflammatory markers in healthy people.

### 2.13. A Randomised Controlled Trial of Thyroglobulin as a Biomarker of Iodine Deficiency: Study Update Ma, Z.F.; Venn, B.; Manning, P.; Skeaff, S.A.

Background: Iodine is required by the thyroid gland to produce thyroid hormones needed for normal growth and development. Thyroglobulin (Tg), a precursor of thyroid hormone, shows promise as a biomarker to assess iodine status; Tg increases in iodine deficiency. Studies in children have found that a median Tg <13 µg/L indicates adequate iodine status. However, there is limited information on the usefulness of Tg to assess iodine status in adults. 

Methods: We are conducting a randomised, placebo-controlled, double blind study of iodine deficient adults aged 18–40 years who consume either a 150 μg of potassium iodate or placebo tablet daily for 24 weeks. Inclusion criteria were: consume ≤2 serving of bread products/day, urinary iodine concentration (UIC) <100 µg/L, and negative for thyroid antibodies. At screening and completion, participants were asked to provide five casual urine samples and a blood sample as well as complete an online food frequency questionnaire. During the intervention, a blood sample was provided every two months. The main outcome of the study is a decrease in Tg concentration from baseline.

Results: To date, 109 participants have been enrolled in the study; 59% were New Zealand European, 7% were Māori and 34% identified as other ethnicities. The median UIC (MUIC) at baseline was 64 μg/L confirming that the participants were mildly iodine deficient (*i.e.*, MUIC of 50–99 μg/L). The mean (±SD) age was 23.6 (3.5) years, 23% were males, and mean (±SD) BMI was 24 (4) kg/m^2^. 

Conclusion: As expected, subjects were mildly iodine deficient at baseline and sociodemographic characteristics in the supplemented and placebo groups were well similar. 

### 2.14. Monitoring the Price Differential between ‘Healthy’ and ‘Less Healthy’ Foods: Key Challenges Mackay, S.

Background: INFORMAS (International Network on Food and Obesity/Non-Communicable Disease Research, Monitoring and Action support) aims to monitor, and benchmark food environments globally. One of the modules proposes a step-wise framework to measure the cost and affordability of population diets. The framework aims to assess the price differential between ‘healthy’ and ‘less healthy’ foods (minimal approach), and diets (expanded approach) and the affordability of ‘healthy’ and ‘less healthy’ diets (optimal approach).

Methods: In order to develop the methodology for the minimal approach, two methods to construct lists of ‘healthy’ and ‘less healthy’ foods were compared: a) pairs of ‘healthy’ and ‘less healthy’ food items, and b) lists of core and discretionary foods. The lists were analyzed using three price metrics. 

Results: Using price per 100 g and price per serve, the ‘healthy’ and ‘less healthy’ items of most pairs were similar in price, and core foods were slightly more expensive than discretionary foods. Using price per kilojoule, ‘healthy’ foods were more expensive than ‘less healthy’ foods. Challenges in developing the food lists were defining commonly consumed foods, defining serve sizes, choosing realistic pairs, ensuring contrast in healthiness between groups, inclusion of fruits and vegetables, inclusion of takeaways and defining an anchor for construction of the lists. 

Conclusion: The expanded approach of comparing ‘healthy’ with current, ‘less healthy’ diets has the potential to overcome many of these limitations.

### 2.15. Acute Dynamic and Compositional Changes in Chylomicrons after a High-Fat Breakfast Meal: Impact of Age Milan. A.; Nuora, A.; Villa, K.; Linderborg, K.; Cameron-Smith, D.

Background: Postprandial lipaemia is described by dynamic changes in size and composition of chylomicrons after a high-fat meal. Differences in chylomicron formation and metabolism affect post-meal lipid clearance and may contribute to cardiovascular and metabolic disease risk. Metabolic and digestive changes associated with ageing may alter lipid metabolism, affecting chylomicron composition and clearance.

Methods: This study examined the differences in chylomicron composition and dynamics between younger and older adults following a high-fat breakfast meal. In a randomised crossover trial, 30 participants aged 20–25 and 60–75 years consumed a high-fat breakfast. Chylomicrons extracted from plasma samples were taken at baseline and for 5 h after the meal. Chylomicron particle size was analysed by dynamic light scattering. Total triacylglyceride (TAG) and apolipoprotein B (ApoB) concentration of chylomicrons were measured by autoanalyser and Western blotting. TAG and phospholipid (PL) species were extracted and analysed by gas chromatography with flame ionisation detection of individual fatty acids. 

Results: Older adults experienced a greater prolonged elevation of chylomicron TAG concentrations and a great number of saturated and monounsaturated fatty acids after the high-fat breakfast compared to younger adults. Younger adults had larger chylomicron particles than older adults after the high-fat breakfast with more PL polyunsaturated fatty acids and lower ApoB concentrations. Older individuals have more, smaller chylomicrons, with an exaggerated TAG response and a greater proportion of monounsaturated fatty acids after a high-fat breakfast compared to younger individuals. 

Conclusion: These data may indicate a mechanism for delayed chylomicron clearance and a less favourable dietary lipid absorption profile in older adults. 

### 2.16. Evaluation of a Nutritional Risk Screening Tool in Children with Cystic Fibrosis Disease Moeeni, V.; Shojaee, P.; Kianifar, H.; Walls, T.; Pattemore, P.; Day, A.

Background: Cystic Fibrosis (CF) is associated with a high risk of malnutrition. The specific aims of this project were to define the nutritional status of children attending two CF clinics (one in Iran, one in New Zealand [NZ]), to compare two nutritional risk screening (NRS) tools and to validate these results against each patient’s full clinical evaluation.

Methods: Sixty nine CF patients (2–18 years) were assessed during consecutive routine outpatient visits over twelve months. Anthropometric measurements were obtained. Both NRS tools were applied to each patient and the results compared to the evaluation of clinical team. The validity of the two NRS scores was assessed in each visit and specificity and sensitivity were calculated.

Results: Under-nutrition was seen only in Iranian children, whereas over-nutrition was more prevalent in NZ patients (*p* = 0.05). The mean sensitivity and specificity for the McDonald tool for all visits were 83% and 78% (Iran) and 75% and 85% (NZ), respectively. The sensitivity and specificity for the Australasian guideline were 67% and 69% (Iran) and 75% and 91% (NZ) respectively. In the Iranian cohort the McDonald tool was able to identify 56%–67% of the patients who had a decline in their BMI centiles.

Conclusions: Both tools successfully recognised patients at risk of malnutrition. The MacDonald tool had comparable sensitivity and specificity to that described previously, especially in Iranian children. This tool may be helpful in recognising at risk CF patients, particularly in developing countries with fewer resources.

### 2.17. Antioxidant Capacities, Total Phenolic and Ascorbic Acid Content of Fruit Available in New Zealand Nguyen, H.V.A.; Mason, S.L.; Savage, G.P.

Methods: Antioxidant capacities, total phenolic and ascorbic acid contents of 40 fruit available in New Zealand were quantified using ABTS and ORAC, Folin-Ciocalteu and titration assays respectively. The fruits contained a wide range of these activities. 

Results: These ranged from 122.3 to 13,631.6 µmol Trolox equivalents (TE)/100 g FW for ORAC; 19.5 to 6045.9 µmol TE/100 g FW for ABTS; 27.4 to 2731.9 mg gallic acid equivalent (GAE)/100 g FW for total phenolic concentrations and 6.2 to 201.3 mg ascorbic acid/100 g FW. Berries showed the highest antioxidant capacities along with high total phenolics and ascorbic acid contents. Both antioxidant capacities, ORAC and ABTS were significantly correlated to the total phenolic concentrations (*p* < 0.001) while no relationship between ascorbic acid and ABTS and only a weak correlation between ascorbic acid and ORAC (*p* < 0.05) were found. The calculated contribution of ascorbic acid to ORAC values was less than 20% for all fruits except green kiwifruit (40.5%), golden kiwifruit (37.5%), tamarillo (29.7%), persimmon (24%) and pineapple (23.4%). 

Conclusion: Together these correlations suggest that phenolic compounds are primarily responsible for the free radical scavenging ability of fruit, while ascorbic acid plays a lesser role.

### 2.18. Salted: Sodium in Commonly Consumed Fast Food in New Zealand Prentice, C.A.; Smith, C.; McLean, R.M.

Background: Sodium intakes in New Zealand are well above current recommendations. Fast foods may be a large contributor to sodium intakes, given that they are typically high in sodium, and are known to be frequently consumed by some population groups. 

Methods: Commonly consumed fast foods were identified from results of the 2008/09 New Zealand Adult Nutrition Survey. The top 12 most commonly consumed fast foods from independent outlets (*n* = 52) were selected to be tested analytically in the laboratory, while the sodium and energy content of all savoury fast foods (16 food types) from large chain restaurants (*n* = 471) were collected from online Nutrition Information Panels. Results were compared with the 2012 United Kingdom Food Standards Agency (UK FSA 2012) sodium concentration targets.

Results: From independent outlets, sausage rolls had the highest concentration of sodium (per 100 g), and a meal of chop suey had the highest sodium per 1000 kJ. Ten of the 12 fast foods tested exceeded the maximum UK FSA 2012 sodium targets. Of the information provided from large chain restaurants, sauces/salad dressings and fried chicken had the highest sodium content. Twelve of the 13 fast foods exceeded the UK FSA 2012 sodium targets. Significant differences (*p* < 0.05) in the sodium content by brand were found for burgers, pizza, sandwiches/wraps, and fries/wedges. 

Conclusion: The majority of commonly consumed fast foods in New Zealand exceeded recommended sodium limits. This study supports the need for a population sodium reduction strategy, which should include fast food reformulation.

Conflict of Interest Statement: Some of the data used in this study were from the 2008/09 New Zealand Adult Nutrition Survey. The New Zealand Ministry of Health funded the survey, and the survey was conducted collaboratively with the University of Otago. The New Zealand Ministry of Health had no role in the design, analysis or writing of this work and the results presented are the work of the authors.

### 2.19. Fatty Acid Profiles of New Zealand Grown and Imported Pine Nuts Savage, G.; Vanhanen, L.; Hider, R.

Background: Pine nuts (*Pinus* spp.) are becoming more popular in New Zealand cuisine and their availability has increased. They have a unique taste because they contain high levels of unsaturated fatty acids. They are an excellent source of dietary fatty acids, such as linoleic and oleic acids. Pine nuts are either locally grown or imported and informal reports suggest that different cultivars have widely different tastes because of the different patterns of fatty acids in the different cultivars. 

Methods: Five different cultivars of pine nuts were harvested from trees growing in Marlborough and the fatty acid profiles were compared to samples of imported pine nuts available in local supermarkets. Individual fatty acids in each sample of nuts were analysed using FastGLC methods and the fatty acids were identified by mass spectrometry. 

Results: Local fresh pine nuts contained high levels of unsaturated fatty acids, oleic and linoleic acid. They also contained several Δ5-olefinic fatty acids, taxoleic, pinolenic, coniferonic, eicosadienoic, sciadonic and eicosatrienoic which are characteristic of gymnosperms. The pattern of these Δ5-olefinic fatty acids can be used to determine the botanical origin of the pine nuts and can assist in the identification of the country of origin. 

Conclusion: Pine nuts are generally assumed to be sourced from European stone pine *(P. pinea*), however, this analysis shows that imported pine nuts are not *P. pinea*. The pinolenic acid content of pine nuts is particularly interesting as it has been shown to have LDL-lowering properties and appears to suppress hunger when eaten.

### 2.20. Carbohydrate Restriction: Are You for Real? Schofield, G.

Public health nutrition practice over the last 50 years has worked towards making recommendations about the types and quantities of foods we should eat. The idea has been to improve health and well-being, and decrease the incidence of the diseases of modern times. In the meantime, the prevalence of these non-communicable diseases has skyrocketed. There are at least three possibilities. First, the guidelines are good, but people don’t understand and/or have any ability to use them. If only people could adhere to the low fat, especially saturated fat guidelines, then population health would be better. This could be because of multiple factors, including but not limited to, the food industry, poor government policy, poor public health communication and poor health systems. The second is that the low fat guidelines are actually not optimal for the control and reduction of chronic diseases and improvements in population health. A third is a combination of these two possibilities. Different dietary approaches can confer health benefits, but those benefits depend on a variety of factors. The current low fat guidelines might do more harm than good, and it is proposed that the use of carbohydrate restriction is a realistic and effective strategy. A more parsimonious theory of metabolic health underpinned by variations in insulin resistance and affected by macronutrient, especially carbohydrate intake, will be presented in this keynote presentation. Last, an alternative set of public health nutrition guidelines, ‘The real food guidelines’, will be presented for consideration.

### 2.21. Low Energy Availability amongst New Zealand Athletes (LEANZ) Study: Proposed Methods Slater, J.; Black, K.; Cooke, R.; Brown, R.

Background: Low energy availability (LEA) occurs when there is insufficient energy for normal physiological functions following exercise. LEA is thought to be common among athletes and can have serious, adverse health implications particularly on reproductive function, hormonal balance and bone health. The prevalence of LEA amongst New Zealand (NZ) athletes is currently unknown, but in other countries has been estimated to be 15.9%.

Methods: To estimate the prevalence of LEA amongst NZ recreational athletes, 146 adult participants were recruited (92 female, 54 male) via gyms throughout NZ. Upon completion of the survey participants were sent a nutrition tip sheet and entered into a prize draw for a grocery voucher. 

Results: Participants completed an online questionnaire comprising of questions from validated eating disorder and LEA questionnaires. Five participants (3.4%) reported a BMI of <18.5kg/m^2^. Eight participants (5.6%) reported a desired BMI of <18.5 kg/m^2^, five of which already fitted into this category. To the question ‘I am preoccupied with the desire to be thinner’, 11 participants (7.5%) responded ‘always’ and eight (5.5%) ‘usually’. Ten females (10.6%) were 15 years or older when they first had their period. Thirty three females were not using oral contraceptive pills of which 15 (45.5%) had their last period over 2 months ago and nine (27.3%) had not had a period for at least six months.

Conclusion: This study should provide important information on the prevalence and predictors of LEA to help with early detection. Therefore, treatment can be implemented before performance and, most importantly, health is compromised.

### 2.22. Pregnant New Zealand Women Are Iodine Deficient despite Government Initiatives to Improve Iodine Status Skeaff, S.; Billing, A.; Chiu, C.; Evans, C.

Background: In response to a re-emergence of iodine deficiency in New Zealand in the 1990s, bread was fortified with iodised salt in 2009 and an iodine supplement of 150 µg/day for pregnant women was recommended in 2010. The aim of this study was to determine the effectiveness of iodine fortification and supplementation to achieve adequate iodine status in pregnant women. 

Methods: A convenience sample of 100 pregnant women was recruited from each of three cities (Dunedin, Hamilton and Wellington) between July 2012 and December 2013. Women were asked to collect a spot urine sample to determine urinary iodine concentration (UIC), and complete a questionnaire to obtain socio-demographic information, iodine supplement use, and frequency of consumption of iodine containing foods. The final sample size was 302 women with a mean (±SD) age of 31(5) years; 13% were of Māori or Pacific Island ethnicity. 

Results: The median UIC of women was 105 µg/L, which falls below the 150–250 µg/L suggested by WHO to indicate adequate iodine status in pregnancy. The median iodine intake from food alone and food with iodised salt was estimated at 76 µg/day and 113 µg/day, respectively; the Estimated Average Requirement in pregnancy is 160 µg/day. Although 65% of women took an iodine supplement, they did so infrequently. Iodine supplementation was a significant predictor of UIC (*p* = 0.048) in multivariate linear regression. 

Conclusion: There has been an improvement in the iodine status of pregnant women (from 38 µg/L in 2005 to 105 µg/L in 2013), but despite government initiatives, pregnant women remain iodine deficient. 

### 2.23. Update of the New Zealand Food Cost Survey: 2014 Smith, C.; Mainvil, L.M.; Walker, L.M.; McPike, L.J.; Gray, E.A.; Parnell, W.R.

Background: Since the 1970s the Department of Human Nutrition at the University of Otago has reported the cost of a basket of food designed to meet the nutritional needs of: adult men and women, adolescent boys and girls (11 to 18 years), children (10, 5, 4 years) and infants (1 year). In 2014 the foods included, and methods used to calculate food costs were revised. 

Methods: Twenty-four hour diet recall data from the New Zealand 2008/09 Adult Nutrition Survey was used to justify the removal or addition of foods. For each food category gram amounts were allocated to sex and age groups to meet New Zealand dietary guidelines. 

Results: The composite diets met energy and macronutrient goals and Nutrient Reference Values (RDI/AI) for all nutrients apart from sodium. Food prices were collected from four supermarkets in Dunedin, Christchurch, Wellington and Auckland in the first week of March. To account for the popularity of individual food items a weighted average of $ per gram was calculated for each food category. In 2014 food costs for an adult male were: Auckland $68, Wellington $69, Christchurch $71, and Dunedin $67. Most of the cost of the basic diet was for meat and poultry, dairy and milk, and fruit and vegetables (all 22%). 

Conclusion: We are confident that the updated Food Cost Survey reflects the food choices of New Zealanders and the basic diet is adequate to meet the nutrient requirements of most. It is important that the affordability of a nutritionally adequate diet is within reach of all. 

### 2.24. In Vitro and in Vivo Prebiotic and Anti-Microbial Activities of Rabbiteye Blueberry Extracts Vuthijumnonk, J.; Heyes, J.A.; Wolber, F.M.; Chua, W-H.; Molan, A.L.

Background: Intestinal microflora plays a crucial role in human health. Their population is modulated in diseases such as cancer. Blueberries contain phytochemicals with biological activity. 

Methods: In the present study, we examined the prebiotic and anti-microbial activities of five rabbiteye blueberry crude extracts *in vitro*. In addition, the *in vivo* activities of two blueberry extracts on intestinal microflora and β-glucuronidase concentration were measured as part of a study of the effects of blueberry extract on 7,12-dimethyl benz[a]anthracene (DMBA)-induced mammary tumorigenesis in a rat model system. 

Results: All five cultivars had prebiotic activity *in vitro* on both *Lactobacillus rhamnosus* and *L. acidophilus*. However, the extracts did not exhibit anti-microbial activity on pathogenic bacteria *E. coli*, *Salmonella typhimurium*, *Staphylococcus aureus* and *Streptococus mutans*. Intestinal microflora DNA was extracted from the caecal contents and caecum mucosal lining of the *in vivo* rat study. Bacterial populations were characterised using real time PCR. DMBA affected bacteria populations (both *Lactobacillus* spp. and *Bifidobacterium* spp.), however, the effect was not significant in *Escherichia coli* populations. Animals gavaged with DMBA and supplemented with blueberry extract contained higher *Lactobacillus* spp. and *Bifidobacterium* spp. populations in comparison with rats which received only DMBA treatment. Interestingly, the *E. coli* population and β-glucuronidase concentration were reduced in rats which received blueberry extract. 

Conclusion: This study suggests that crude extracts of rabbiteye blueberry exhibit prebiotic activity both *in vitro* and *in vivo*. Although no anti-microbial effect of the extracts was observed *in vitro*, a positive effect was found *in vivo*. 

### 2.25. The Effect of a Beverage Containing Fruit Fibre and Fruit Polyphenols, Either Individually or Together, on Gut Health Wallace, A.J.; Eady, S.L.; Hunter, D.C.; Skinner, M.A.; Huffman, L.; Ansell, J.; Blatchford, P.; Wohlers, M.; Herath, T.D.; Hedderley, D.; Rosendale, D.; Stoklosinski, H.; McGhie, T.; Sun-Waterhouse, D.; Redman, C.

Background: This pilot study examined the effect of a Boysenberry beverage (750 mg polyphenols), an apple fibre beverage (7.5 g dietary fibre) and a Boysenberry plus apple fibre beverage (750 mg polyphenols and 7.5 g dietary fibre) on gut health. 

Methods: The trial beverages were 350 mL taken in two doses every day. Twenty-five individuals completed the study. The study was a placebo-controlled crossover study where every individual consumed one of the four treatments in turn. Each treatment phase was for four weeks’ duration and was followed by a two-week washout period where no trial beverages were consumed. 

Results: There were no differences in faecal concentrations of total bacteria, *Bacteriodes-Prevotella-Porphymonas* group, *Bifidobacterium* spp., *Clostridium perfingens* and *Lactobacillus* spp. among any of the treatment groups. Short chain fatty acid concentrations did not vary between treatment groups either, but PGE_2_ concentrations were higher after the Boysenberry beverage. A daily gut health questionnaire was also completed by participants. People reported an increase in beneficial effects relating to gut health such as less flatulence and bloating, after consuming the Boysenberry treatment in comparison with the baseline, the apple fibre and the Boysenberry plus apple fibre drink (*p* < 0.001). People reported generally better mental well-being after each washout period, placebo and the apple fibre juice than after the Boysenberry juice and the Boysenberry plus apple fibre juice drink (*p* = 0.05). 

Conclusion: Apple fibre juice drinks may make people feel better even though there were no objective improvements in gut health.

## 3. Affiliations

- Ansell, J., The New Zealand Institute for Plant and Food Research Ltd, Palmerston North, New Zealand
- August, C.R., Department of Human Nutrition, University of Otago, Dunedin, New Zealand
- Bacon, C.J., Department of General Practice and Primary Health Care, University of Auckland, New Zealand
- Billing, A., Department of Human Nutrition, University of Otago, Dunedin, New Zealand
- Black, K., Department of Human Nutrition, University of Otago, Dunedin, New Zealand
- Blatchford, P., Plant and Food Research, Palmerston North, New Zealand
- Bolland, M.J., Department of Medicine, Faculty of Medical and Health Sciences, University of Auckland, New Zealand
- Brown, R., Department of Human Nutrition, University of Otago, Dunedin, New Zealand
- Burrows, L., Department of Human Nutrition, University of Otago, Dunedin, New Zealand
- Butler, A., Canterbury District Health Board, Christchurch, New Zealand
- Cameron-Smith, D., Liggins Institute, University of Auckland, Auckland, New Zealand
- Chepulis, L.M., School of Nursing, Faculty of Health, Education and Humanities, Waiariki Institute of Technology, Rotorua, New Zealand
- Chisholm, A., Department of Human Nutrition, University of Otago, Dunedin, New Zealand
- Chiu, C., Department of Human Nutrition, University of Otago, Dunedin, New Zealand
- Chua, W.-H., Institute of Food, Nutrition and Human Health, Massey University, Palmerston North, New Zealand
- Cooke, R., Department of Human Nutrition, University of Otago, Dunedin, New Zealand
- Crowe F.L., Biosecurity Science, Food Science and Risk Assessment, Ministry for Primary Industries, Wellington, New Zealand
- Day, A., Department of Paediatrics, University of Otago, Christchurch, New Zealand
- Dyall, L., Department of General Practice and Primary Health Care, University of Auckland, New Zealand
- Eady, S.L., New Zealand Institute for Plant and Food Research Limited, Lincoln, New Zealand
- Ellet, S., Discipline of Nutrition, Auckland University, New Zealand; Nutrigenomics New Zealand, Auckland University, New Zealand
- Evans, C., Department of Human Nutrition, University of Otago, Dunedin, New Zealand
- Ferguson, L.R., Discipline of Nutrition, Auckland University, New Zealand; Nutrigenomics New Zealand, Auckland University, New Zealand
- Fleming, E.A., Department of Human Nutrition, University of Otago, Dunedin, New Zealand
- Francis, E., School of Nursing, Faculty of Health, Education and Humanities, Waiariki Institute of Technology, Rotorua, New Zealand
- Gray, E.A., Department of Human Nutrition, University of Otago, Dunedin, New Zealand
- Greer, J., Department of Human Nutrition, University of Otago, Dunedin, New Zealand
- Haeusler S., Diabetes Research, Capital and Coast District Health Board, Wellington, New Zealand
- Han, D.Y., Discipline of Nutrition, Auckland University, New Zealand; Nutrigenomics New Zealand, Auckland University, New Zealand
- Hang, D.T., Faculty of Animal Husbandry and Veterinary Medicine, Hue University of Agriculture and Forestry, Hue City, Viet Nam
- Hathaway, S., Biosecurity Science, Food Science and Risk Assessment, Ministry for Primary Industries, Wellington, New Zealand
- Hayman, K.J., Department of General Practice and Primary Health Care, University of Auckland, New Zealand
- Hedderley, D., Plant and Food Research, Palmerston North, New Zealand
- Herath, T.D., The New Zealand Institute for Plant and Food Research Ltd, Palmerston North, New Zealand
- Heyes, J.A., Institute of Food, Nutrition and Human Health, Massey University, Palmerston North, New Zealand
- Hider, R., Food Group, Department of Wine, Food and Molecular Biosciences, Lincoln University, Lincoln, Canterbury, New Zealand
- Huffman, L., Plant and Food Research, Palmerston North, New Zealand
- Hunter, D.C., Plant and Food Research, Mount Albert, Auckland, New Zealand
- Jackson, R.T., Section of Epidemiology and Biostatistics, School of Population Health, Faculty of Medical and Health Sciences, University of Auckland, Auckland, New Zealand
- Jesuthasan, A., Discipline of Nutrition, Auckland University, New Zealand; Nutrigenomics New Zealand, Auckland University, New Zealand
- Kepa, M., Department of General Practice and Primary Health Care, University of Auckland, New Zealand
- Kerse N., Department of General Practice and Primary Health Care, University of Auckland, New Zealand
- Kianifar, H., Department of Paediatrics, Mashhad University of Medical Sciences, Mashhad, Iran
- Krebs J.D., Diabetes Research, Capital and Coast District Health Board, Wellington, New Zealand
- Laing, B., Discipline of Nutrition, Auckland University, New Zealand; Nutrigenomics New Zealand, Auckland University, New Zealand
- Linderborg, K., Food Chemistry, Department of Biochemistry, University of Turku, Turku, Finland
- Loc, N.T., Faculty of Animal Husbandry and Veterinary Medicine, Hue University of Agriculture and Forestry, Hue City, Viet Nam
- McGhie, T., Plant and Food Research, Palmerston North, New Zealand
- Mackay, S., School of Population Health, University of Auckland, New Zealand
- McLean, R.M., Department of Human Nutrition, University of Otago, Dunedin, New Zealand; Department of Preventive and Social Medicine, Dunedin School of Medicine, University of Otago, New Zealand
- McPike, L.J., Department of Human Nutrition, University of Otago, Dunedin, New Zealand
- Ma, Z.F., Department of Human Nutrition, University of Otago, Dunedin, New Zealand
- Mainvil, L.M., Department of Human Nutrition, University of Otago, Dunedin, New Zealand
- Manning, P., Department of Medicine, University of Otago, Dunedin, New Zealand
- Marlow, G., Discipline of Nutrition, Auckland University, New Zealand; Nutrigenomics New Zealand, Auckland University, New Zealand
- Mason, S.L., Wine, Food and Molecular Biosciences Department, Lincoln University, New Zealand
- Massarotto, C., The New Zealand Institute for Plant and Food Research Ltd, Palmerston North, New Zealand
- Milan. A., Liggins Institute, University of Auckland, Auckland, New Zealand
- Miller, J., Biosecurity Science, Food Science and Risk Assessment, Ministry for Primary Industries, Wellington, New Zealand
- Mirosa, M., Department of Food Science, University of Otago, Dunedin, New Zealand
- Moeeni, V., Department of Paediatrics, University of Otago, Christchurch, New Zealand
- Molan, A.L., Department of Biology, College of Sciences, Diyala University, Diyala, Iraq
- Morgenstern, M., The New Zealand Institute for Plant and Food Research Ltd, Lincoln, Christchurch, New Zealand
- Moyes, S.A., Department of General Practice and Primary Health Care, University of Auckland, New Zealand
- Nguyen, H.V.A., Food Technology Department, International University, Ho Chi Minh City, Viet Nam
- Nuora, A., Food Chemistry, Department of Biochemistry, University of Turku, Turku, Finland
- Parnell, W.R., Department of Human Nutrition, University of Otago, Dunedin, New Zealand
- Parry-Strong A., Diabetes Research, Capital and Coast District Health Board, Wellington, New Zealand
- Pattemore, P., Department of Paediatrics, University of Otago, Christchurch, New Zealand
- Pearson, K., Department of Human Nutrition, University of Otago, Dunedin, New Zealand
- Prentice, C.A., Department of Human Nutrition, University of Otago, Dunedin, New Zealand
- Price, M., Canterbury District Health Board, Christchurch, New Zealand
- Redman, C., Plant and Food Research, Palmerston North, New Zealand
- Reid, J., Biosecurity Science, Food Science and Risk Assessment, Ministry for Primary Industries, Wellington, New Zealand
- Rosendale, D., Plant and Food Research, Palmerston North, New Zealand
- Savage, G.P., Food Group, Department of Wine, Food and Molecular Biosciences, Faculty of Agriculture and Life Sciences, Lincoln University, Canterbury, New Zealand
- Schofield, G., Sports Performance Research Institute, AUT University, Auckland, New Zealand
- Shojaee, P., Department of Paediatrics, Mashhad University of Medical Sciences, Mashhad, Iran
- Skeaff, S., Department of Human Nutrition, University of Otago, Dunedin, New Zealand
- Skinner, M.A., Plant and Food Research, Mount Albert, Auckland, New Zealand
- Slater, J., Department of Human Nutrition, University of Otago, Dunedin, New Zealand
- Smith, C., Department of Human Nutrition, University of Otago, Dunedin, New Zealand
- Spence, H., Department of Human Nutrition, University of Otago, Dunedin, New Zealand
- Stoklosinski, H., Plant and Food Research, Palmerston North, New Zealand
- Sun-Waterhouse, D., Plant and Food Research, Mount Albert, Auckland, New Zealand
- Teh, R., Department of General Practice and Primary Health Care, University of Auckland, New Zealand
- Tey, S.L., Clinical Nutrition Research Centre, Singapore Institute for Clinical Sciences, A*STAR, Singapore
- Tra, T.T.T., Faculty of Animal Husbandry and Veterinary Medicine, Hue University of Agriculture and Forestry, Hue City, Viet Nam
- Tuan, L.M., Faculty of Hospitality and Tourism, Hue University, Hue City, Viet Nam
- Vanhanen, L., Food Group, Department of Wine, Food and Molecular Biosciences, Lincoln University, Lincoln, Canterbury, New Zealand
- Venn, B., Department of Human Nutrition, University of Otago, Dunedin, New Zealand
- Villa, K., Food Chemistry, Department of Biochemistry, University of Turku, Turku, Finland
- Vuthijumnonk, J., Institute of Food, Nutrition and Human Health, Massey University, Palmerston North, New Zealand
- Walker, L.M., Department of Human Nutrition, University of Otago, Dunedin, New Zealand
- Wallace, A.J., New Zealand Institute for Plant and Food Research Limited, Lincoln, New Zealand
- Walls, T., Department of Paediatrics, University of Otago, Christchurch, New Zealand
- Wohlers, M., Plant and Food Research, Mount Albert, Auckland, New Zealand
- Wolber, F.M., Institute of Food, Nutrition and Human Health, Massey University, Palmerston North, New Zealand

